# Rationale and design of a type 2 diabetes prevention intervention for at-risk mothers and children at a Federally Qualified Healthcare Center: EPIC El Rio Families Study Protocol

**DOI:** 10.1186/s12889-021-10392-w

**Published:** 2021-02-12

**Authors:** David G. Marrero, Robert M. Blew, Kelly N. B. Palmer, Kyla James, Denise J. Roe, Melanie D. Hingle

**Affiliations:** 1 University of Arizona Health Sciences Center for Border Health Disparities, 1295 North Martin Ave., P.O. Box 210202, Tucson, AZ 85721 USA; 2 Department of Nutritional Sciences, College of Agriculture & Life Sciences, The University of Arizona, 1177 E. 4th Street, Shantz Building, Room 328, Tucson, AZ 85721 USA; 3 University of Arizona Collaboratory for Metabolic Disease and Prevention, Abrams Public Health Center, 3950 S. Country Club Rd, Tucson, AZ 85714 USA; 4 Family and Child Wellness at El Rio Community Health Center, 450 W Paseo Redondo, Tucson, AZ 85701 USA; 5 Department of Epidemiology and Biostatistics, Mel and Enid Zuckerman College of Public Health, The University of Arizona, 1295 N. Martin Ave., P.O. Box 245210, Tucson, AZ 85724 USA

**Keywords:** Diabetes, gestational, Mothers, Diabetes mellitus, type 2, Life style, Body weight, Child, Primordial prevention, Preventive health

## Abstract

**Background:**

Exposure to gestational diabetes mellitus (GDM) is associated with increased risk for type 2 diabetes (T2DM) in mothers, and poor cardiovascular health among offspring. Identifying effective methods to mitigate T2DM risk has the potential to improve health outcomes for mothers with a history of GDM and their children. The goal of the EPIC El Rio Families Study is to implement and evaluate the effects of a 13-week behavioral lifestyle intervention on T2DM risk factors in at-risk mothers and their 8- to 12-year-old children. We describe herein the rationale for our specific approach, the adaption of the DPP-based curriculum for delivery to patients of a Federally Qualified Health Center (FQHC), and the study design and methodology.

**Methods:**

The effects of the intervention on reduction in excess body weight (primary outcome), hemoglobin A1c, blood pressure, and changes in lifestyle behaviors associated with weight trajectory and T2DM risk in mother-child dyads will be evaluated during a 13-week, group randomized trial wherein 60 mothers and their children will be recruited to the intervention or wait-listed control conditions at one of two FQHC locations. Intervention participants (*n* = 30) will begin the group program immediately, whereas the wait-listed controls (*n* = 30) will receive a booklet describing self-guided strategies for behavior change. Associated program delivery costs, acceptability of the program to participants and FQHC staff, and potential for long-term sustainability will also be evaluated.

**Discussion:**

Successful completion in our aims will produce a scalable program with high potential for replication and dissemination, and estimated intervention effects to inform T2DM prevention efforts on families who use the FQHC system. The results from this study will be critical in developing a T2DM prevention model that can be implemented and scaled across FQHCs serving populations disproportionately burdened by T2DM.

**Trial registration:**

ClinicalTrials.gov NCT03781102; Date of registration: 19 December 2018.

**Supplementary Information:**

The online version contains supplementary material available at 10.1186/s12889-021-10392-w.

## Background

Type 2 diabetes (T2DM) continues to be a major public health problem in the United States, with over 30 million persons afflicted and estimates projecting a continued increase [1, 2]. Youth comprise a small yet growing number of new T2DM cases, with significant increase in overall incidence observed between 2002 and 2015 [3]. Major advances have been made toward understanding risk factors for T2DM and pathophysiology in youth. Dissemination of family-focused T2DM prevention programs to community settings remains rare, and a majority of programs have not directly involved parents or focused on youth who are at greatest risk. Thus, prior efforts have often resulted in diffuse interventions and insufficient implementation of lifestyle changes at home, as parents exert significant influence over the home diet and physical environment and opportunities [4–10]. The paucity of effective T2DM prevention programs adapted for delivery to at-risk families in accessible, affordable, and safe settings remains a critical barrier to reducing population prevalence and risk.

Herein we describe a study designed to address gaps specific to T2DM prevention for women with a history of gestational diabetes mellitus (GDM) and/or prediabetes and their 8- to 12-year-old children, who are nearly twice as likely to develop T2DM before the age of 22 compared to youth with no maternal GDM diagnosis [11]. This intervention study, *Encourage, Practice, and Inspire Change in El Rio Families* (*EPIC El Rio Families*), will engage those at greatest risk for T2DM, provide evidence-based intervention content and strategies to support lifestyle behavior modification, and leverage extant medical infrastructure and personnel for program delivery to address key social determinants of health as well as maximize future program impact and sustainability in the home [12, 13].

The goal of the *EPIC El Rio Families* project is to implement and evaluate the effects of a behavioral lifestyle intervention on T2DM risk factors in at-risk mothers and their children delivered within a Federally Qualified Health Center (FQHC) by trained FQHC personnel. Leveraging an FQHC network serving 110,000 underinsured and uninsured patients for intervention delivery provides an opportunity to take an efficacious T2DM program to scale. The FQHC participating in this study serves those among the highest risk for T2DM in the Southwestern region of the United States: low-income women previously diagnosed with gestational diabetes mellitus (GDM) or who have confirmed pre-diabetes (HbA1c of 5.7–6.4%), and their child, aged 8- to 12-years-old, who by virtue of genetics, pre- and post-natal exposures, and weight status, are also at significantly increased T2D risk [14, 15].

Up to 10% of pregnancies are affected by GDM, and the prevalence of GDM is increasing among pregnant women as obesity continues to increase [16, 17]. Among Hispanic women of Mexican origin, GDM is more common than non-Hispanic whites and its prevalence has increased since 2006 [18, 19]. In addition to the increased T2DM risk experienced by women with a history of GDM, there is growing evidence that maternal obesity and GDM are contributing to the increase in obesity and T2DM in their children [20, 21]. In the SEARCH for Diabetes in Youth study, intrauterine exposure to maternal diabetes and obesity was attributed to T2DM in 47.2% of the adolescent cohort [3], indicating that exposure to GDM is a strong predictor of T2DM development. Additionally, it is now known that hereditary risk for prediabetes and T2DM is impacted by common genetic variations associated with risk for developing β-cell dysfunction affecting insulin sensitivity [21]. These genetic factors combined with increasing levels of obesity-related insulin resistance are central components in T2DM development. Collectively, these facts enable us to convey to mothers who have had GDM that their children have an increased risk for developing T2DM, especially if excessive weight gain is not prevented.

There is now considerable evidence that lifestyle modification interventions that promote modest weight loss and increased physical activity can substantially reduce the risk for developing T2DM in adults [22–24]. There remains, however, a dearth of rigorously tested T2DM prevention lifestyle strategies for high-risk youth. This is due, in part, to the relatively low prevalence of T2DM in adolescents when compared with adults, and the complexity and costs associated with conducting such trials. In the absence of a definitive T2DM prevention study with youth at increased risk, lifestyle modification to support healthy eating and physical activity, thereby optimizing weight gain trajectories and insulin sensitivity, is the generally accepted mechanism to decreasing risk. In this context, the family-based behavioral approach is the standard model for preventing and treating childhood obesity and associated health risks. Indeed, prior research has established that interventions featuring parents as primary change agents had better outcomes to traditional child-only focused approaches [25–29] and offer potential for sustainability and cost-effectiveness.

We contend that mothers at increased risk for T2DM in combination with their child should be the focus of an intervention to decrease familial T2DM risk, and further, that counseling mothers about the T2DM health risks in combination with strategies to reduce these risks increase the likelihood of sustained changes to lifestyle behaviors and the home environment that will support risk reduction. We hypothesize that the delivery of a T2DM prevention program emphasizing lifestyle modifications in families to reduce weight, improve diet quality, increase physical activity, and manage stress for high-risk mothers would also benefit their children.

## Methods/design

### Adaptation of a diabetes prevention curriculum for *EPIC El Rio Families*

The intervention duration, content, and activities, designed to support families in meeting national recommendations were drawn from the intervention literature [30, 31] and our previous work [32–34]. Intervention “dose” (duration × time) was modeled after the successful adult-focused Diabetes Prevention Program [35] and the 2017 U.S. Preventive Services Task Force (USPSTF) evidence-based recommendations for weight loss, behavior change, and cardio-metabolic risk reduction in youth [36].

*EPIC El Rio Families* is intended for families served by El Rio Community Health Center (hereafter, El Rio), a Federally Qualified Health Center in the Southwestern United States serving more than 110,000 underinsured and uninsured patients, and a research partner in this study. El Rio patient demographics (85% Hispanic or Latina (84% White, 16% American Indian), 15% non-Hispanic (80% White, 11% Black, 6% Asian, 2% Pacific Islander, 1% American Indian); more than 90% will live at or below the federal poverty level) and our formative work [37] suggest that the study sample will be majority-Hispanic, of whom at least one-third will prefer to participate in Spanish-only groups. Prior to implementation, language and cultural adaptations of intervention content were guided by focus groups conducted with women patients of El Rio [37]. Findings from focus groups prompted translation of all materials into Spanish, delivery of the intervention by bicultural, bilingual coaches, and culturally-sensitive diet and physical activity recommendations that do not violate cultural beliefs and practices.

Intervention sessions will be group-based, attended by up to 15 mother-child dyads, and led by a minimum of two trained bilingual FQHC health and wellness staff. Each session is approximately 2 h in length and was designed for delivery over 16 consecutive weeks at one of two El Rio locations. Total duration was shortened to 13 weeks following formative research to accommodate school and holiday scheduling demands, with optional booster sessions following the core 13-week program.

The intervention content is focused on practical strategies for modifying the behaviors associated with the pathogenesis of T2DM [38], including availability and accessibility of nutrient- and calorie-dense foods and food preparation strategies in the home, reducing intake of sugar-sweetened beverages, increasing time spent in physical activity and decreasing time spent in sedentary activities, stress management, and obtaining quality sleep (Table 1). Participants will be engaged in learning through interactive food demonstrations, energy balance activities (e.g., label reading, shopping preparation and planning), and physical activities appropriate for the entire family. Sessions provide repeated opportunities to practice healthy lifestyle behaviors. All activities incorporate evidence-based behavior change techniques and behavioral targets [39, 40]. Each session follows a similar format consisting of the following five components: a featured physical activity encouraging families to get moving upon arrival; small group discussions focused on goal-setting and building intra- and inter-family camaraderie; food demonstration and tasting opportunities centered around increasing dietary fiber (vegetables, whole grains, and legumes) and reducing added sugar intake; group activities to increase foundational knowledge and skills related to healthy food selection, physical activity benefits, and creating a supportive home environment; and, opportunities to set new weekly goals or revise previous goals. Mothers and children are divided into two groups, with children engaged in games-based physical activity while mothers engage in discussions with program leaders and other participants regarding implementation of realistic, effective plans for family lifestyle behavior change and the use of proactive food and physical activity parenting practices [41]. Every third session, a behavioral health specialist will lead stress management exercises for all family members.

**Table 1:** EPIC El Rio Families Program

Session	Session Topics
0	What to expect/meet your coaches; setting goals
1	Basic diabetes prevention; energy density of foods
2	Making healthy food available and accessible in the home; how to prepare and enjoy vegetables, whole grains, legumes
3	Swap screen time for active time; what is physical activity and why is it important?
4	Serve just the right amount of food to keep body weight healthy; MyPlate
5	Have more fun staying active as a family; benefits of family physical activity
6	Enjoy calmer, healthier, more relaxed meals; making mealtime family time
7	How to choose tasty, low-kcal beverages and drink less sugary drinks; label reading
8	Learn and practice healthy sleeping habits and manage stress
9	Eating out and making healthy choices; problem-solving
10	Increase the variety of physical activity; overcome barriers to being active
11	Making family physical activity happen; problem-solving
12	Talking back to negative thoughts
13	Finding the best food for you at your grocery store; body positivity

To reflect what will be the standard operating procedures for future implementation and expansion of the program, El Rio will select individuals to become lifestyle intervention coaches following extant procedures used by the organization. Criteria for consideration include recommendation by supervisors or colleagues, previous experience and comfort level working with children and families, organizational skills, and commitment to coaching participants through 13 consecutive weeks of programming. Coaches are also encouraged to become certified in group fitness and health coaching and receive instruction on core nutrition concepts related to T2DM prevention. This approach reflects how El Rio already implements and trains staff to conduct their adult weight management and T2DM treatment programs.

Select staff are then trained to be lifestyle coaches using the approach developed by the EPIC El Rio Families Study MPI and used by the CDC National DPP [35, 42]. This 3-day training process has been successfully used to train hundreds of lifestyle coaches [43] using standardized training materials including a comprehensive lifestyle coach manual. The manual will be modified to reflect new elements of the curriculum specifically designed for *EPIC El Rio Families* and include a section to support FQHC staff who wish to conduct additional trainings to help ensure standardized program delivery.

### Evaluation of the *EPIC El Rio Families* Study

This study uses a group randomized design to compare two groups of mothers who have had GDM or have been diagnosed with prediabetes, along with their children, who are between 8- and 12-years-old. The goal is to recruit 60 mother/child dyads to either the EPIC El Rio Families intervention or wait-listed control conditions. To identify potential participants with histories of GDM and/or prediabetes who are patients at El Rio, we will query the FQHC’s electronic health record (EHR). Women with a history of GDM during any of their pregnancies and/or a prior diagnosis of prediabetes and who have children 8- to 12-years-old at time of study will be contacted by phone and mail and invited to participate in *EPIC El Rio Families*. The participating child must be a biological child but does not have to be the direct product of a pregnancy with GDM. With attention to the composition of the FQHC patient population, the study sample will reflect majority Hispanic women from lower income groups.

**Fig. 1:**
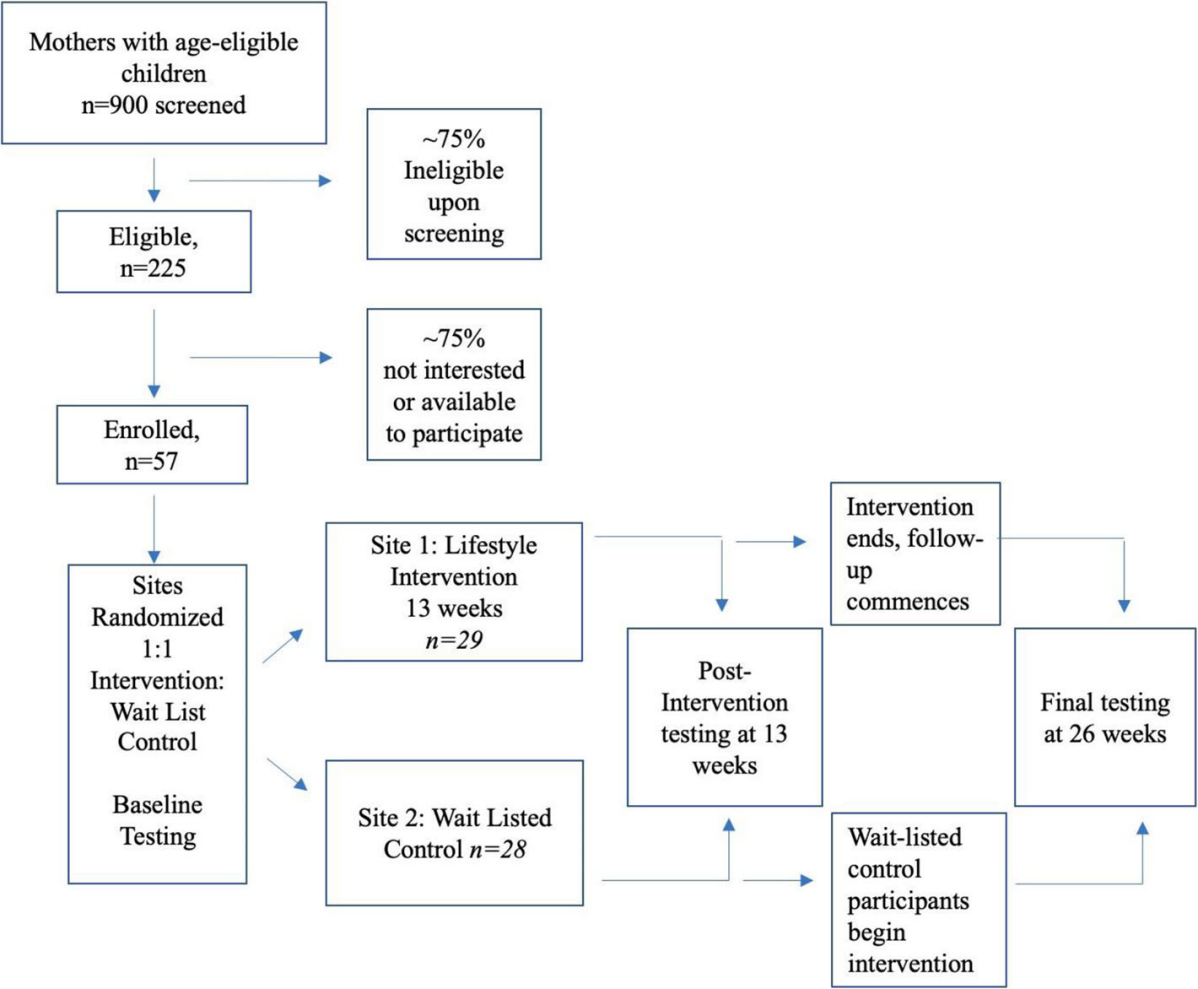
EPIC El Rio Families Participant Anticipated Enrollment and Study Design

Two El Rio clinics (A and B) have been selected to participate in this pilot study. Randomization to the intervention and wait-list control conditions will be performed *by site* using flip of a coin. Randomization by site has the least potential for contamination during the initial delivery period. Upon confirming respondent eligibility and obtaining written informed consent/assent, the research coordinator will match participants to the intervention site geographically nearest to their home, assign a study ID, and schedule baseline measurements. Following measurements, intervention participants will begin the 13-week face-to-face group program immediately, whereas the wait-listed controls will receive information about the offer to participate in the intervention after the first implementation and that they will continue to receive standard of care including access to nutritional counseling if desired. After 13 weeks, the intervention participants will transition to a follow-up phase while the wait-listed controls will begin the intervention (Fig. 1). Blinding is not possible with this design.

This design is responsive to standards of care for T2DM prevention [44] and will provide the opportunity to investigate pre−/post-intervention effects, intervention versus control effects, and maintenance effects. The number of participants and design also allow us to evaluate processes critical to successful implementation by El Rio, informing future replication and the potential scaling of the intervention to other sites in this and other FQHC networks. This clinical trial (ClinicalTrials.gov identifier NCT03781102) has been approved by the University of Arizona Institutional Review Board and all participants will provide informed consent or assent prior to participation.

Changes in percent weight for the mothers and changes in the BMI z-score for the children are used to confirm that there is adequate statistical power with the sample size of *n* = 60 dyads. For mothers, the calculation is based on the observed change in the DEPLOY study for both sexes [34]. The mean change in body weight after 6 months was − 6.0% (standard deviation = 4.0%). For children, the calculation is based on data from the EPIC Kids Study in which the mean change in BMI z-score was 0.054 (standard deviation = 0.02) [32]. The power calculation assumed that in each period (intervention versus wait-list control) there will be 3 groups with 10 dyads per group (*n* = 30 intervention versus *n* = 30 wait-list control). The statistical power is > 80% for the mothers and 95% for the children assuming an intra-group correlation or 0.15 or below (two-sided alpha level of 0.05). As this is a pilot study to provide estimates for a larger trial, there is no adjustment for multiple comparisons.

The primary outcome is percent change in body weight and BMI at 13 weeks (post-intervention) and 26 weeks (follow-up/weight maintenance). Body weight and height will be measured using a calibrated, digital scale with a mechanical height rod and a stadiometer. Adult BMI is categorized using international classifications of BMI (overweight, 25–29.9 kg/m^2^; obese, > 30 kg/m^2^) [45]. In children, BMI percentile is determined using age- and sex-specific growth charts developed by the CDC in 2000 [46]. Lacking a gold standard for measuring change in weight status in children, we will use the recommended BMI z-score change, which has been established as a good proxy for fat mass z-score change [47]. Child and adult waist circumference, linked to metabolic syndrome in both populations, will be measured at the umbilicus. All measures will be completed in duplicate, and the average of the two measures will be used. Secondary physiological outcomes in children and mother**s** include changes in blood pressure and hemoglobin A1c (HbA1c). These data will be collected using portable, CLIA approved technologies. The definition of hypertension will be adjusted for age per national guidelines [48, 49].

.

Secondary behavioral outcomes associated with weight trajectory and T2DM risk in children and mothers will be assessed at 13 weeks (post-intervention) and 26 weeks (follow-up/weight maintenance). Child dietary intake will be assessed with *two, nonconsecutive*, interviewer-administered *24-h dietary recalls* conducted telephonically by trained nutritionists and entered into the Nutrient Data System for Research (Minneapolis, MN, v. 2012) [50]. Overall diet quality and its components (total vegetables, whole fruit, whole grains, plant, animal, and seafood proteins, fats, sodium, sugar, and refined grains) will be calculated using the Healthy Eating Index-2015, a valid and reliable measure of diet quality designed to assess the degree to which an individual’s intake conforms to dietary recommendations [51, 52]. Child moderate to vigorous physical activity and sedentary behavior will be assessed using the *Youth Activity Profile,* a self-administered 7-day (previous week) recall questionnaire validated for use in children ages 8- to 12-years-old [53] that includes 15 items divided into three sections: activity at school, activity out-of-school, and sedentary. Sleep behavior will be assessed using the *Children’s Sleep Habits Questionnaire, Abbreviated*, a 22-item survey designed for parent report of key child sleep domains that encompass major medical and behavioral sleep disorders in school-aged children [54]. Maturity will be assessed from a self-report of Tanner’s breast/genital and pubic hair descriptions. The validated questionnaire presents illustrations of developmental stages shown to agree with pubertal staging by a physician [55].

Parental dietary intake will be assessed using the validated *Arizona Food Frequency Questionnaire*, a self-administered semi-quantitative 159-item questionnaire in which respondents report how often they typically consume specific foods and food groups and associated portion sizes in the past 3 months [56, 57]. Parent physical activity will be assessed using the validated *Arizona Activity Frequency Questionnaire*, a self-administered 59-item questionnaire in which participants report whether they performed each activity during the past 28 days [58]. Primary outputs include daily energy expenditure (kilocalories), number of hours per day spent in each activity, and number of activities reported for each category.

The home food and physical activity environment will be assessed using the *Family Nutrition and Physical Activity Tool,* a 21-item survey of the family home environment and practices associated with children’s risk of becoming overweight. Parents will use the tool to report the frequency of breakfast and family meals, modeling of nutrition, nutrient dense foods and high calorie beverages, restriction and reward, parent modeling physical activity, child’s physical activity, screen time, TV in bedroom, and sleep routines [59, 60].

Given the considerable impact of social and environmental factors on participants’ ability to follow through with health recommendations, we will collect demographic and socioenvironmental data associated with T2DM risk and health disparities (e.g., food insecurity, housing stability, culture and language, community support, socioeconomic status, financial barriers, literacy and numeracy) using the PRAPARE questionnaire developed for use in health care settings, and already routinely implemented by El Rio. All surveys will be made available in English and Spanish.

The intention-to-treat principle will be used for all the analyses. Differences in participants’ baseline characteristics between the two groups will be evaluated by a two-sample *t*-test for continuous outcomes and by Chi-square test or Fisher’s exact test for categorical outcomes. Initial analyses will compare the changes between baseline and follow-up between the intervention versus wait-list control groups using two-sample sample *t*-tests. Subsequently, a mixed-model analysis of covariance including treatment group, time, and baseline value of the outcome will be used to assess the effects of intervention at 13 and 26 weeks. Participants will be treated as a random effect nested within group. An interaction effect between time and treatment group will be assessed first. If there is no interaction effect, the overall treatment difference will be assessed. Otherwise, the treatment difference will be assessed at each time point. In order to measure the relative cost-effectiveness of these programs, we will also explore costs associated with implementation by El Rio staff.

Given the feasibility focus of this study, we will also evaluate whether aspects of the intervention work as intended, including participant *acceptability* (participant satisfaction) and program *relevance* (rated using brief surveys of the relevance of the intervention to daily life, promoters and barriers to program attendance and engagement, the degree to which families report using the intervention to guide behavioral choices, and surveys of lifestyle coaches trained to deliver the intervention). We will assess *adoption* by El Rio and *integration with the existing clinical setting* using semi-structured interviews with El Rio wellness staff and administrators and *feasibility* (of delivery by providers and FQHC) by examining recruitment, enrollment, and retention rates, session attendance data, and observed participant engagement with intervention activities. *Fidelity* will be assessed through observation of research staff following an established rubric, and *program costs* will be evaluated using a bottom-up micro-costing approach (including direct medical and non-medical costs including personnel gross hourly salaries, intervention material costs, and overhead costs related to use of facilities for prevention services will be tracked and analyzed in partnership with El Rio’s data team members). We will also explore the *potential for replication and dissemination* using semi-structured interviews with El Rio wellness staff assigned to coordinate and deliver the program, and administrators and advisory board members who understand how to align the intervention with El Rio’s fiscal and strategic plans, and who have relationships with other FQHCs in Arizona and nationally. Our a priori focus on factors influencing *sustainability* (e.g., reach, integration with other health/wellness programs, institutionalization) beyond the research funding period will remain a major consideration throughout the proposed study.

## Discussion

There is a growing need to develop and evaluate prevention initiatives that focus on lifestyle modification for youth and their families at highest risk for T2DM. We will, in collaboration with an FQHC, create a T2DM risk-reducing intervention program (*EPIC El Rio Families*) for delivery to mothers at high risk for T2DM because of their history of GDM and/or prediabetes, and who have school-aged children who are also at risk. The intervention is unique in that it was designed at its inception to integrate with the clinical operation of an FQHC and developed in partnership with client-facing health and wellness FQHC staff. FQHCs by their nature are well suited to implement interventions for populations who have historically experienced significant barriers to accessing quality health care. Past diabetes prevention studies implemented in medical centers were efficacy trials that gave minimal consideration to the cost of implementation [22–24]. These research-oriented programs were then adapted to be used in community settings [35]. As a result, they are often not optimized for dissemination in large volume primary care settings. Moreover, very few have simultaneously targeted mothers and their children at risk for T2DM. By designing the program to be implemented in an extant FQHC facility that cares for families, we will be able to answer several questions that are crucial to developing an effective translation model that can be applied across the FQHC system. The results from this study will be critical in developing a T2DM prevention model that can be implemented and scaled across FQHC settings to effectively mitigate this burgeoning problem in medically underserved youth and adult. In this context, using the FQHC system to implement diabetes prevention programs in populations that have an increased burden from T2DM is an important public health initiative.

## Supplementary Information

**Additional file 1.**

**Additional file 2.**

## Data Availability

Not applicable.

## Abbreviations

EPIC: Encourage, Practice and Inspire Change
DPP: Diabetes Prevention Program
GDM: Gestational diabetes mellitus
T2DM: Type 2 diabetes mellitus
FQHC: Federally Qualified Health Center
CDC: Centers for Disease Control and Prevention
YMCA: Young Men’s Christian Association
CLIA: Clinical Laboratory Improvement Amendments
HbA1c: Hemoglobin A1c
BMI: Body Mass Index
