# Supplementary material for: Rationale and design of a type 2 diabetes prevention intervention for at-risk mothers and children at a Federally Qualified Healthcare Center: EPIC El Rio Families Study Protocol
Source: BMC Public Health. 2021 Feb 12;21:346. doi: 10.1186/s12889-021-10392-w (PMC7881686; doi:10.1186/s12889-021-10392-w)
Supplement: Supplementary file 1 — Additional file 1. [file 12889_2021_10392_MOESM1_ESM.docx]

**Data and Safety Monitoring Plan |** Investigator: Hingle, MD | Application: NIH 1 R34 DK118486-01

The proposed study meets the NIH definition of clinical research, although it is not a Phase III clinical trial. The required data and safety monitoring plan is described herein. Our plan will anticipate a range of possible outcomes and responses. Key components will include a communication protocol to support interaction among the investigators and research team, parent and child participants, regulatory authorities (IRB), and the NIH.

*Entities conducting monitoring of the study:* The Human Subjects Committee (institutional Review Board [IRB]) at the University of Arizona (UA) will review and provide oversight as IRB of record for the proposed research.

*What is monitored:* Once approved by the UA IRB, the following aspects of the study and study conduct are monitored: all procedures to ensure conformity with the approved study protocol; unforeseen circumstance that might arise and affect participant safety; all reports of serious adverse events and defined in 38 CFR 46 (death, new or prolonged hospitalization, persistent or significant disability or incapacity; congenital anomaly or birth defect); other significant adverse events that lead to participant dropout, participant termination by the principal investigators, or termination of participation in the intervention.

*Data handling and quality:*

*University of Arizona*

Assessments will be conducted with study participants as described in the Research Strategy and Protection of Human Subjects sections of this application. All aspects of data collection and data storage will be carefully monitored to ensure rapid detection or errors, inconsistencies or other problems. Study personnel involved in data collection will follow a strict written protocol that describes study measures for protecting data privacy, clearly explains to study participants that they have the right to refuse to participate or refuse to answer any individual question that they wish to not answer, and emphasizes reporting as accurately and truthfully as possible. The Principal Investigators and Co-Investigator are experienced in training study staff in handling sensitive and confidential data, and in the storage and processing of such data.

Physical access to the data center is restricted to authorized personnel. All servers are housed in a locked rack and secured from the Internet and other university departments through the use of a hardware-based firewall and virtual LAN. A host-based firewall is installed and configured on each server as a secondary level of defense from outside intrusion. All servers and computer systems on the network are configured with domain-managed accounts and password controls with audit logs. Access to server resources such as project data are restricted to authorized users only as approved by the project's Principal Investigators.

All servers and data are backed up daily with secure off-site storage at the University of Arizona's Computer Center.

*El Rio Community Health Center*

All personally identifiable information within the El Rio Electronic Health Record is – and will remain - protected and secure. El Rio has established a policy and procedure regarding the Privacy of Protected Health Information (PHI) and Security of Electronic Protected Health Information (EPHI).  All EHRs are HIPAA compliant, and use the highest quality processes based on ISO13485, ISO 9001 and SSAE16 standard during software design and development.

*Adverse events:*

We will use the definition of adverse events in the NIH OHRP document “Guidance on Reviewing and Reporting Unanticipated Problems Involving Risks to Subjects or Others and Adverse Events.” Adverse event (AE): Any untoward or unfavorable medical occurrence in a human subject, including any abnormal sign (for example, abnormal physical exam or laboratory finding), symptom, or disease, temporally associated with the subject’s participation in the research, whether or not considered related to the subject’s participation in the research.

Serious adverse event (SAE): Any adverse event that: (1) results in death; (2) is life-threatening (places the subject at immediate risk of death from the event as it occurred); (3) results in inpatient hospitalization or prolongation of existing hospitalization;(4) results in a persistent or significant disability/incapacity; (5) results in a congenital anomaly/birth defect; or (6) based upon appropriate medical judgment, may jeopardize the subject’s health and may require medical or surgical intervention to prevent one of the other outcomes listed in this definition.

*Reporting:* It is highly unlikely that any of the study procedures will cause an AE or SAE. Should an AE or SAE occur, study staff are trained to immediately report AEs connected to implementation of the intervention to PIs Hingle and Marrero who will keep a log of AEs and SAEs, and El Rio Co-I Mockbee, a medical doctor. In the event of an SAE, we will file a report with the University of Arizona IRB (who will be the IRB of record). As part of this process, the University of Arizona IRB will determine if the event is directly related to project procedures, so that It can be determined if project procedures should be modified. Information about all AEs and SAEs will be provided in annual progress reports to University of Arizona IRB, El Rio IRB, and the NIH.
