# Supplementary material for: Rationale and design of a type 2 diabetes prevention intervention for at-risk mothers and children at a Federally Qualified Healthcare Center: EPIC El Rio Families Study Protocol
Source: BMC Public Health. 2021 Feb 12;21:346. doi: 10.1186/s12889-021-10392-w (PMC7881686; doi:10.1186/s12889-021-10392-w)
Supplement: Supplementary file 2 — Additional file 2. [file 12889_2021_10392_MOESM2_ESM.docx]

**Dissemination Plan | Investigator: Hingle, MD | Application: NIH 1 R34 DK118486-01**

NIH policy requires that all clinical trials be registered and submit results to clinicaltrials.gov (<https://grants.nih.gov/grants/guide/notice-files/NOT-OD-16-149.html>). Please remember that the journals require that registration be complete prior to enrollment of the first participant. The plan can be brief, but at a minimum it must contain sufficient information to assure that:

--the applicant will ensure that clinical trials under the award are registered and results information is submitted to ClinicalTrials.gov as outlined in the policy and according to the specific timelines stated in the policy

--informed consent documents for the clinical trial(s) will include a specific statement relating to posting of clinical trial information at ClinicalTrials.gov

--the recipient institution has an internal policy in place to ensure that clinical trials registration and results reporting occur in compliance with policy requirements

In 2018, the Office for Human Research Protections at the University of Arizona implemented the same definition of a clinical trial as the NIH. (Please find the UA policy here: <https://rgw.arizona.edu/sites/default/files/clinical_trials.pdf>) We will meet all corresponding regulatory requirements for clinical trials supported by the NIH <https://grants.nih.gov/policy/clinical-trials.htm>, including: registering the trial information on ClinicalTrials.gov prior to beginning the study, posting a paragraph in the informed consent notifying participants that we plan to post the protocol and results, and how to request this information from us. We will submit a clinicaltrials.gov registration number as part of our submission of the full IRB application, we will use a single IRB of record (University of Arizona IRB), and we will submit a copy of the IRB approved consent form posted in clinicaltrials.gov or similarly accepted and designated publicly available federal repository no later than 60 days after the last study visit by any subject.

We will also submit relevant updates and summary results to ClinicalTrials.gov throughout the study, and make this information public through our postings, meetings with participants, and our Federally Qualified Health Center partner (El Rio Community Health Center). The investigators have already completed Good Clinical Practice and Responsible Conduct of Research trainings and will ensure all study staff and students do the same prior to proposed start date for the project. As the study commences, we will revise our registry to include relevant updates (recruitment rates, retention rates, feasibility metrics) and will submit summary results and make this information publicly available as previously described.
